# Origin and Spread of the Dengue Virus Type 1, Genotype V in Senegal, 2015–2019

**DOI:** 10.3390/v13010057

**Published:** 2021-01-04

**Authors:** Idrissa Dieng, Marielton dos Passos Cunha, Moussa Moïse Diagne, Pape Mbacké Sembène, Paolo Marinho de Andrade Zanotto, Ousmane Faye, Oumar Faye, Amadou Alpha Sall

**Affiliations:** 1 Arboviruses and Haemorrhagic Fever Viruses Unit, Virology Department, Institut Pasteur de Dakar, Dakar BP 220, Senegal; Idrissa.DIENG@pasteur.sn (I.D.); MoussaMoise.DIAGNE@pasteur.sn (M.M.D.); Ousmane.FAYE@pasteur.sn (O.F.); Amadou.SALL@pasteur.sn (A.A.S.); 2 Department of Animal Biology, Faculty of Science et Technics, Université Cheikh Anta Diop de Dakar(UCAD), Fann BP 5005, Dakar, Senegal; mbacke.sembene@ucad.edu.sn; 3 Laboratory of Molecular Evolution and Bioinformatics, Department of Microbiology, Biomedical Sciences Institute, University of São Paulo, São Paulo 05508-000, Brazil; marieltondospassos@gmail.com (M.d.P.C.); pzanotto@usp.br (P.M.d.A.Z.); 4 BIOPASS (IRD-CBGP, ISRA, UCAD), Campus de Bel-Air, BP 1386, Dakar CP 18524, Senegal

**Keywords:** dengue virus type 1, phylogeny, phylogeography, origin, Senegal

## Abstract

Dengue virus (DENV) is the most widespread arthropod-borne virus, with the number and severity of outbreaks increasing worldwide in recent decades. Dengue is caused by genetically distinct serotypes, DENV-1–4. Here, we present data on DENV-1, isolated from patients with dengue fever during an outbreak in Senegal and Mali (Western Africa) in 2015–2019, that were analyzed by sequencing the envelope (E) gene. The emergence and the dynamics of DENV-1 in Western Africa were inferred by using maximum likelihood and Bayesian methods. The DENV-1 grouped into a monophyletic cluster that was closely related to those from Southeast Asia. The virus appears to have been introduced directly into Medina Gounass (Suburb of Dakar), Senegal (location probability = 0.301, posterior = 0.76). The introduction of the virus in Senegal occurred around 2014 (95% HPD = 2012.88–2014.84), and subsequently, the virus moved to regions within Senegal (e.g., Louga and Fatick), causing intense outbreaks in the subsequent years. The virus appears to have been introduced in Mali (a neighboring country) after its introduction in Senegal. In conclusion, we present evidence that the outbreak caused by DENV-1 in urban environments in Senegal and Mali after 2015 was caused by a single viral introduction from Asia.

## 1. Introduction

The dengue virus (DENV-1–4) is the world’s most important arbovirus transmitted by infected *Aedes* mosquitoes. It is endemic in more than 100 countries, and it is estimated that 50 million cases occur annually worldwide [1,2]. Its vector, the *Aedes aegypti* mosquito, is widely distributed in tropical and subtropical areas around the world [3]. DENV is an enveloped, positive-sense, single-stranded RNA virus that belongs to the genus *Flavivirus*, family Flaviviridae [4,5]. Its genome has a single open reading frame encoding for three structural proteins (capsid (C), membrane (M) and envelope (E)) and seven non-structural (NS) proteins (NS1, NS2A, NS2B, NS3, NS4A, NS4B and NS5) [6]. Dengue viruses have four antigenically and genetically distinct serotypes, sharing around 65% of genome similarity [7], subsequently subdivided into distinct genotypes [8,9,10]. Dengue virus infection has diverse clinical manifestations, which range from asymptomatic illness to hemorrhagic fever that can culminate in death [11].

Since the first half of the 20^th^ century, all serotypes experienced an increase in genetic diversity [12], causing variations leading to new genotypes with distinct lineages [13,14]. Some studies have revealed associations between genetic diversity and features such as clinical manifestations, virulence and epidemic potential [8,15]. DENV-1 exists in five distinct genotypes (genotypes I–V) [16,17]. Recently, a basal group of DENV-1 with highly divergent sequences was reported, possibly constituting a new genotype (genotype VI) [16].

In Africa, the landscape of DENV circulation was historically dominated by the occurrence of sylvatic DENV-2 [18]. Over the past two decades, a drastic change in epidemiological patterns has been observed in Africa, with urban outbreaks of DENV reported in Senegal [19] and several cases exported to Europe [20]. In Senegal, DENV-1 was first isolated from two cases in 1979 (Digoutte J.P., personal communication). Since then, no studies reported isolation of this serotype. In 2015, a study on malaria in children less than 10 years old allowed for the detection of three DENV-1 cases in Guediawaye (Medina Gounass), in the suburb of Dakar [21]. This was followed two years later by an outbreak, mainly of DENV-1, in Louga city with 131 confirmed cases (submitted paper).

In 2018, another outbreak associated with DENV-1 occurred in Fatick city, Senegal. Since the notification of the first confirmed cases in the Fatick region on 21 September 2018, and as of 27 October 2018, a total of 1740 suspected cases were reported. Among them, 145 were confirmed, and the remaining 1595 were not characterized as DENV-1. Seven districts from four regions have continued to report confirmed cases: Touba (Diourbel region, 105 cases), Diourbel (Diourbel region, 1 case), Mbacke (Diourbel region, 1 case), Fatick (Fatick region, 33 cases), Gossas (Fatick region, 1 case), Coki (Louga region, 1 case) and Richard-Toll (Saint Louis region, 3 cases) [22].

Despite the increased number of sporadic cases and outbreaks in Senegal during the last 10 years, the origin and spread pattern of circulating DENV-1 strains remain unknown. At present, no phylodynamics studies of dengue virus (DENV-1–4) in Senegal have been reported. The present study describes the origin and phylogeographic profile of the DENV-1 epidemiologic spread in Senegal from 2015 to 2019.

## 2. Materials and Methods

### 2.1. Ethics Committee

Samples used in this study are part of the Institut Pasteur in Dakar collection (WHO Collaborating Centre for Arboviruses and/or Hemorrhagic Fever Reference and Research). None of the data were directly derived from human samples, but rather from a cell culture supernatant. Therefore, all the samples were anonymous and only isolation IDs were used during the analysis.

### 2.2. Sample Collection

**Figure 1:**
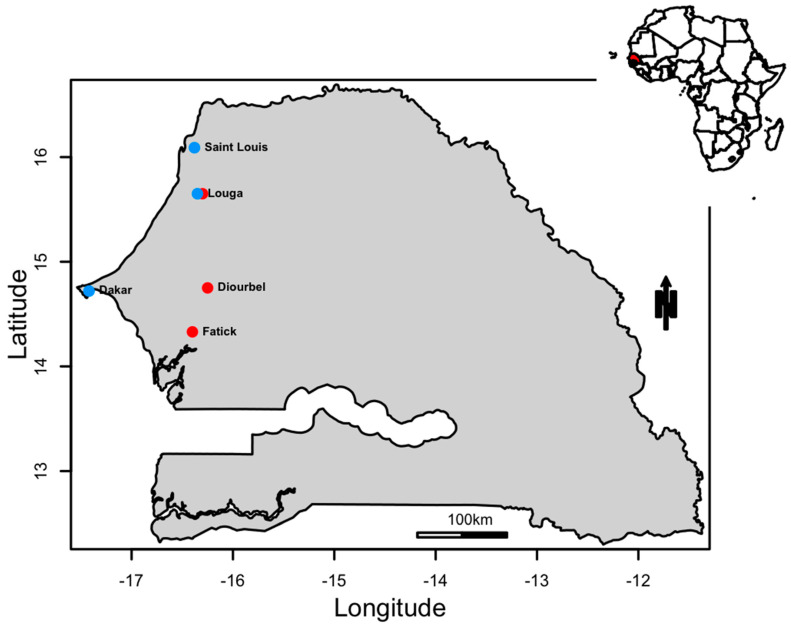
A map showing the locations where DENV−1 positive samples were sampled. The red dots correspond to outbreak samples and the blue dots to sporadic samples.

The strains analyzed during this study were obtained from a cell culture supernatant derived from sporadic DENV cases from 2015 to 2019 and epidemic strains sampled between 2017 and 2018 (Figure 1). Throughout the study, 189 samples were subjected to quantitative reverse transcription PCR (RT-qPCR) to determine serotypes using a TibMolBiol Modular Dx Dengue typing kit (Cat-No. 40-0700-24) (TibMolBiol, Berlin, Germany). Forty-seven were positive for DENV-1, and, of these, a total of 36 isolates from 6 different localities across Senegal and Mali were selected (Table 1) and included in the present study. All other samples belonged to the other serotypes (DENV-2 and DENV-3). No co-infections were detected.

### 2.3. Virus Isolation

Virus isolation was attempted for samples determined to be positive by RT-qPCR. Two hundred microliters of the sample was diluted 1:10 in Leibovitz’s L-15 medium (L15) and added to a 25 cm^2^ flask over a monolayer of C6/36 (*Aedes albopictus*) cell line at 80% confluence, followed by an incubation at 28 °C for 1 h to allow virus adsorption. After incubation, the L15 medium containing 5% FBS, 1% penicillin streptomycin and 0.05% Fungizone was added into a flask and incubated for 10 days or until observation of a cytopathic effect (CPE). To assess viral infection, indirect immunofluorescence (IFA) was conducted as previously described [23]. The flask content was transferred into a 15 mL tube and clarified by low-speed (2500 rpm) centrifugation at 4 °C for 5 min. The supernatant was harvested and stored at −80 °C until further use.

### 2.4. Molecular Characterization

Viral RNA was extracted from 140 μL of cell culture supernatants using a QIAamp viral RNA kit (Qiagen, Hilden, Germany) according to the manufacturer’s instructions. The extracted RNA was eluted in 60 μL of elution buffer and placed in ice for further use. For cDNA synthesis, 10 μL of viral RNA was mixed with 1 μL of the random hexamer primer (2 pmol), and the mixture was heated at 95 °C for 2 min. Reverse transcription was performed in a 20 μL mixture containing a mix of 2.5 U RNasin (Promega, Madison, WI, USA), 1 μL of desoxynucleotide triphosphate (dNTP) (10 µM each dNTP) and 5 U of AMV reverse transcriptase (Promega, Madison, WI, USA) and incubated at 42 °C for 60 min. PCR products were generated using the set of primers D1E1F/D1E1R, D1E2F/D1E2R and D1E3F/D1E3R [24], which amplify the overlapping fragment of the full E gene region. Five microliters of cDNA was mixed with 10× buffer, 3 μL of each primer, 5 μL of dNTPs (10 µM), 3 μL of MgCl_2_ and 0.5 μL of Taq polymerase (Promega, Madison, WI, USA).

### 2.5. Sequencing and Genome Assembly

The obtained amplicons were purified using a QIAquick Spin PCR Purification kit (Qiagen, Hilden, Germany) and then sent for bidirectional sequencing with an ABI 377 automated sequencer (Applied Biosystems, Foster City, CA, USA) to GENEWIZ Services using the same PCR primers. The raw data were then sent to the laboratory for analysis. Chromatograms were analyzed using CodonCode Aligner 3.7.1 (Codon Code, Center Ville, MA, USA) with a Phred quality score cut off of 20 as the cut-off for low-quality sequence trimming.

### 2.6. Data Sets

The complete E gene sequences available for DENV-1 with information regarding the location and year of isolation were recovered from the National Center for Biotechnology Information (NCBI) (https://www.ncbi.nlm.nih.gov/genbank/) website in GenBank format and later converted into FASTA format (sequences available until April 2019). To determine which genotypes and lineages circulated in Senegal and were associated with the outbreak, we resampled the genotypes with large numbers of sequences based on the amount of sequences available for the genotypes that were less sampled (mean of the low sampled genotypes + standard deviation). The genotypes were resampled using the Decrease Redundancy tool hosted at ExPASY (https://www.expasy.org/genomics), with a maximum number of sequences of 5 (*n* = 5). As a result, we obtained non-redundant representative sequences (*n* = 59), named dataset-1 (Table S1). Based on the results of the phylogenetic characterization of dataset-1, the outbreak appears to be caused by the monophyletic DENV-1 group in the Southeast Asian clade. To reconstruct the origin and the virus spread in Senegal (Western Africa), we constructed a resampled dataset using all the sequences from the Southeast Asian clade, which included sequences isolated from viruses in different regions of Asia (East Asia, South Asia, Southeast Asia and Western Asia), Africa (Central Africa, East Africa and Southern Africa) and Oceania. Sequences were initially resampled, excluding all identical sequences, following two criteria: (*i*) when identical from the same location, we kept the oldest sequence (https://biopython.org/wiki/Sequence_Cleaner), and (*ii*) when identical from more than one distinct location, we kept the sequence in by location following the first criterion (https://www.expasy.org/genomics). In the end, all distinct locations/countries were resampled, including a maximum of 5 sequences per location. The African sequences characterized in this paper were isolated between 2015 and 2019 and combined with re-sampled sequences from a Southeast Asian clade, named dataset-2 (*n* = 94) (Table S2). All the datasets were aligned using Clustal Omega v.1.2.1 [25]. Recombinant sequences were screened using all the algorithms implemented in the RDP4 program (RDP, GENECONV, BootScan, MaxChi, Chimaera, SiScan and 3 Seq) with default standard settings [26]. The alignment of recombinant free sequences was manually inspected and edited using the program AliView v.1.18.

### 2.7. Phylogenetic Analysis

Viral phylogenies based on E gene sequences for all the datasets were estimated using the maximum likelihood (ML) phylogenetic approach implemented in IQ-TREE v.1.5.5 software [27] with automatic model selection conducted by ModelFinder using the Bayesian information criterion (BIC) [28]. The robustness of the tree topology was tested during 1000 non-parametric bootstrap analyses. The final tree was visualized and plotted using FigTree v.1.4.3 (http://tree.bio.ed.ac.uk). All sequences used in this work are presented in the following format: genotype/accession number/strain name/locale of isolation/date of isolation.

### 2.8. Discrete Phylogeographic Inference

We explored the temporal signal (i.e., molecular clock structure) and data quality of dataset-2 with TempEst v.1.5.3 [29]. The spatio-temporal spread of the DENV-1 outbreak in Senegal and Mali was reconstructed under a Bayesian framework implemented in BEAST v.1.10.4 [30] using the general time-reversible model with the gamma-distributed rate variation substitution model (GTR + G), as described by the Akaike information criterion (AIC) in jModelTest v.2.1.10. Based on previous estimates of evolutionary dynamics of related DENV-1, we tested for an uncorrelated relaxed molecular clock, assuming a log-normal distribution, in combination with three non-parametric population growth models: (*i*) the standard Bayesian skyline plot (BSP; 10 groups), (*ii*) the Bayesian skyride plot and (*iii*) the Bayesian skygrid model (Table S3). Phylogeographical patterns and parameters were estimated running the Markov chain Monte Carlo (MCMC) for 50 million states and sampling every 50,000 states with 10% burn-in. MCMC convergence obtained after reaching an effective sample size (ESS) >200 was examined with Tracer v.1.7.1 [31]. Likewise, the maximum clade credibility (MCC) tree was visualized and edited with FigTree v.1.4.4 (http://tree.bio.ed.ac.uk). To calculate the log marginal likelihood for the molecular clock and demographic model selection, we used the path sampling (PS) and stepping-stone (SS) sampling approaches by running 100 path steps of 1 million iterations each.

### 2.9. Selection Analysis

We analyzed the changes observed in the DENV-1 samples we sequenced, comparing them to the MH679991 from Singapore, using the mixed effects model of evolution (MEME) [32]. We assumed that positive selection for each site can be inferred when the β^+^ parameter that informs the rate of nonsynonymous substitutions (dN) is greater than α, which informs the rate of synonymous substitutions (dS) using the Datamonkey Adaptive Evolution Server (http://datamonkey.org).

## 3. Results

In order to gain insight about DENV-1 circulation in Western Africa, we investigated the origin, the mode of spread and the transmission dynamics of DENV-1 circulating in Senegal between 2015 to 2019 (Figure 1) using viral samples obtained across Senegal and Mali (Table 1).

For this study, a total of 36 samples of DENV-1 (Table 1) confirmed by RT-qPCR were amplified, sequenced and aligned with DENV-1 E gene sequences available from GenBank.

The sequences were 1485 nucleotides in length, and no evidence for recombination was found. Synapomorphic amino acid substitutions were observed in 10 out of the 495 sites when the African sequences where compared with the Singapore sequence (the sequence isolated in the Southeast Asian region). Among the amino acid changes, eight were non-conservative and two were conservative (Table 2). Most of the amino acid changes, with the exception of codon site 444, had had β^+^ > α, suggestive of directional selection, as inferred by the mixed effects model of evolution (MEME).

**Figure 2:**
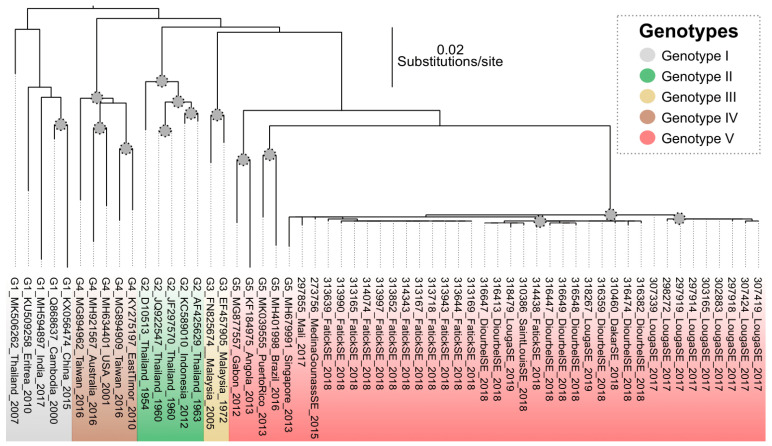
Maximum likelihood viral genealogy inferred using the sequences from Senegal and Mali and strains representative of described genotypes. Grey circle on nodes represent bootstrap values greater than 75. The scale bar represents the number of substitutions per site.

The maximum likelihood viral genealogy based on dataset-1 revealed that the sequenced strains belonged to a monophyletic group within genotype V. These isolates were associated with sequences from Singapore, Brazil and Puerto Rico (Figure 2).

**Figure 3:**
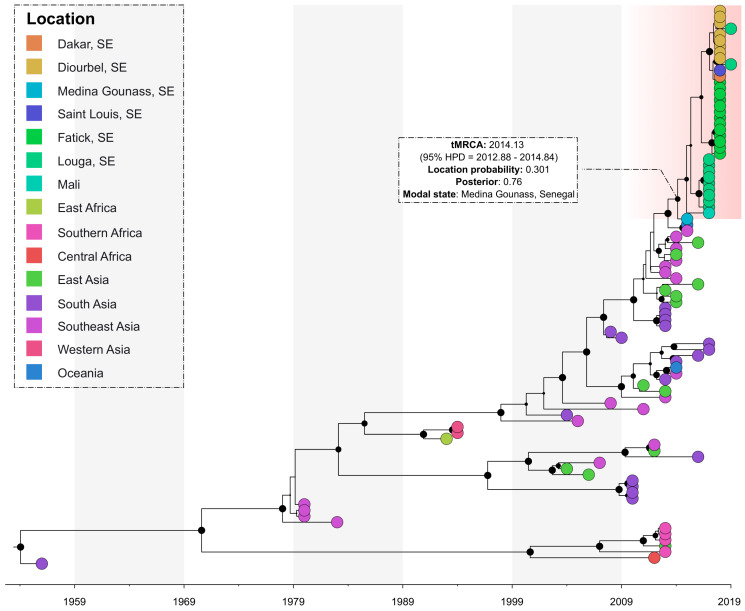
Bayesian discrete phylogeography of dengue strains isolated in Senegal between 2015–2019. Senegalese strains are grouped in a monophyletic cluster (highlighted in red). The black marbles in the nodes represent the posterior probability value and their size is proportional to their value.

The phylogeographic analysis of the sequences showed that all the sequenced viruses originated from the Southeast Asian region and were introduced in Medina Gounass, Senegal (location probability = 0.301, posterior = 0.76). It also suggests that a single introduction possibly occurred around 2014.13 (95% HPD = 2012.88–2014.84) and then dispersed to several other regions across the country (i.e., Louga, Fatick, Diourbel and Dakar) and to the neighboring country of Mali (Figure 3).

## 4. Discussion

The epidemiological pattern of DENV in Africa has changed during the last two decades. This shift has been highlighted by the reports of dengue virus outbreak in all regions across the African continent and/or cases exported to Europe [20]. Between 1970 and 2000, the dengue emergence in Senegal, as in many African countries, was mainly characterized by the circulation of sylvatic DENV-2 with sporadic cases notably in rural areas [18,33,34]. In 2009, a shift appeared with an unprecedented urban DENV-3 epidemic with 196 confirmed cases out of 696 suspected cases across four important cities (i.e., Louga, Fatick, Dakar and Thies) [19]. Since then, between 2017 and 2018, the regular circulation of DENV-1 and DENV-3 occurred in big cities in Senegal [22].

Despite the increased number of DENV cases in Senegal, there were no scientific studies on its origin and dynamics in the region. To our knowledge, this study is the first to elucidate the phylogeny and phylogeography of DENV in Senegal. The amino acid change analysis of sequenced isolates, compared to the Singapore strain (MH679991), identified eight non-conservative amino acid changes located at positions 412, 414, 415, 420, 428, 432, 433 and 444, whereas those at positions 435 and 440 were conservative (Table 2). Although we did not obtain a significant *p*-value for the codon site analyses, sites 305, 129, 50, 3 and 11 had high values of *β*^+^ compared to *α*, which is suggestive of directional, positive selection, possibly imposed by either humans, vectors or both. The diversification of DENV has been observed over time, and its possible diverse implications, from immune cross-reaction among serotypes to vector competence, have to be taken into account. The phylogenetic inference revealed that the sequenced strains belonged to a monophyletic cluster of genotype V, which likely originated in Asia in the 1940s–1950s [17,35]. This particular genotype is known to be circulating in the Americas and Africa and includes some sequences from West Africa [17,36]. Importantly, genotype V experienced the largest geographic expansion in Africa [37,38]. This was probably followed by its subsequent diversification among a susceptible local population after its introduction from Asia [17].

The phylogeographic reconstruction showed the virus was imported to Medina Gounass, Senegal (location probability = 0.301, posterior = 0.76) from the Southeast Asian region. This region was shown to be an important hub with a pivotal role in the global diffusion of DENV-1 [17] and an important source of dengue virus epidemics across the world [39]. The introduction of DENV-1 in Senegal occurred around 2014.13 (95% HPD = 2012.88–2014.84), and subsequently, the virus moved to regions across the country and neighboring Mali, causing multifocal outbreaks and sporadic cases in the subsequent years [22].

In addition, several serological surveys were conducted, mainly in the Kedougou region (southeastern Senegal), to assess the impact of DENV amplification on human populations. In 1981, 11% of children were positive for antibodies against DENV-2. In addition, positive serological responses were found yearly from 1982 to 1985, particularly in 1984 with 3% positive sera among those tested. IgM antibodies to DENV-2 were detected in November 1988 and November 1991 in 4.6–5.7% and 0.8% of individuals examined, respectively. Authors in [40] reported a high prevalence of the Zika virus antibody during a seroprevalence study in the Dielmo area (located in the Fatick region where a DENV outbreak occurred in 2018). All these previous studies, combined with the frequent molecular detection of other dengue serotypes in Senegal [19,41,42], have stressed that there is a risk of more severe dengue outbreaks in the future, manifesting antibody-dependent enhancement (ADE) as is the case for regions with serotype co-circulation [43]. Our study is the first to report the spatio-temporal dynamics of DENV-1 in Senegal. It highlights the importance of continuous molecular surveillance of arthropod-borne viruses since the spread of emerging pathogens occurs rapidly among distant locations. We hope that this work will constitute a reference for future DENV phylodynamic studies in Senegal. Like Senegal, many other African countries have reported dengue outbreaks [44,45,46], but unfortunately did not provide published sequences. This lack of crucial information begs for large-scale sampling to elucidate the dynamics of DENV across the continent.

The global expansion and recent DENV outbreaks around the world are likely a result of anthropogenic degradation, including unplanned urbanization causing local environmental changes that are resulting in the persistence of the main vector in tropical and subtropical areas [2]. With increasing vector infestation levels, the movement of symptomatic patients, asymptomatic patients and infected vectors may promote the spread of the virus to other localities and ultimately pose a significant risk of its continuous dissemination [10,47]. The increase of DENV activity in Medina Gounass, located in the capital, Dakar, where major financial and administrative activities take place, as well as the increased number of dengue outbreaks in Senegal are clear indications of a serious public health threat with a great potential for economic and public health impacts. Therefore, it is crucial to understand viral movement and to identify transmission hot-spots to allow for both intensive vector surveillance and the adequate design of disease control and prevention strategies [48].

## Figures and Tables

**Table 1:** Sequenced strains during this study.

Sequence ID	City	Country	Year	Epidemiological Context
307339	Louga	Senegal	2017	Outbreak
307424	Louga	Senegal	2017	Outbreak
303165	Louga	Senegal	2017	Outbreak
302883	Louga	Senegal	2017	Outbreak
307419	Louga	Senegal	2017	Outbreak
273756	Dakar	Senegal	2015	Sporadic
297855	NA	Mali	2017	Outbreak
310386	Saint Louis	Senegal	2018	Sporadic
310460	Dakar	Senegal	2018	Sporadic
313165	Fatick	Senegal	2018	Outbreak
313167	Fatick	Senegal	2018	Outbreak
313169	Fatick	Senegal	2018	Outbreak
313639	Fatick	Senegal	2018	Outbreak
313644	Fatick	Senegal	2018	Outbreak
313718	Fatick	Senegal	2018	Outbreak
313852	Fatick	Senegal	2018	Outbreak
313943	Fatick	Senegal	2018	Outbreak
313990	Fatick	Senegal	2018	Outbreak
313997	Fatick	Senegal	2018	Outbreak
314074	Fatick	Senegal	2018	Outbreak
314343	Fatick	Senegal	2018	Outbreak
314438	Fatick	Senegal	2018	Outbreak
316359	Diourbel	Senegal	2018	Outbreak
316382	Diourbel	Senegal	2018	Outbreak
316413	Diourbel	Senegal	2018	Outbreak
316447	Diourbel	Senegal	2018	Outbreak
316474	Diourbel	Senegal	2018	Outbreak
316548	Diourbel	Senegal	2018	Outbreak
316647	Diourbel	Senegal	2018	Outbreak
316649	Diourbel	Senegal	2018	Outbreak
318267	Louga	Senegal	2019	Sporadic
318479	Louga	Senegal	2019	Sporadic
297914	Louga	Senegal	2017	Outbreak
297918	Louga	Senegal	2017	Outbreak
297919	Louga	Senegal	2017	Outbreak
298272	Louga	Senegal	2017	Outbreak

**Table 2:** Observed codon site substitutions including MH679991 from Singapore. Column 4, Except Isolates (Amino Acid), indicates the sequenced isolates during this study, which show a different amino acid at a given position compared to the remaining; the observed amino acid at this position is indicated in parenthesis. Although not significant at a *p*-value > 0.05, the mixed effects model of evolution (MEME) suggests that most codon sites had *β*^+^ > *α*, with the exception of codon site 444.

Position	MH679991 (Singapore)	Sequenced Strains during this Study	Except Isolates (Amino Acid)	*β^+^*	*α*
3	C	C	314438(S)	28.01	0.07
11	F	F	314438(Y)	16.45	0.00
50	V	V	307424(A)	136.69	0.06
129	I	I	310460(T)	137.29	0.00
305	S	S	313990(P)	144.25	0.00
412	R	M	−	4.233	0.15
414	T	I	−	5.45	0.00
415	R	L	−	7.25	0.00
418	T	T	314438(I)	7.62	0.03
420	G	W	−	5.10	0.03
428	G	V	−	5.07	0.00
432	G	V	−	5.07	0.00
433	E	G	−	9.29	0.00
435	W	L	−	4.40	0.00
440	L	F	−	2.89	0.00
444	N	Y	−	5.75	20.09

## Supplementary Materials

The following are available online at https://www.mdpi.com/1999-4915/13/1/57/s1, Table S1: Dengue virus 1 Envelope gene sequences used in the genotype phylogenetic analysis, Table S2: Dengue virus 1 Envelope gene sequences used in the phylogeographic and phylodynamics analysis, Table S3: Model comparison of strict molecular clock and demographic growth models through path sampling (PS) and stepping stone (SS) methods. Bold numbers indicate the best fitting model
