# Supplementary material for: Origin and Spread of the Dengue Virus Type 1, Genotype V in Senegal, 2015–2019
Source: Viruses. 2021 Jan 4;13(1):57. doi: 10.3390/v13010057 (PMC7824722; doi:10.3390/v13010057)
Supplement: Supplementary file 1 [file viruses-13-00057-s001.pdf]

Table S1. Dengue virus 1 Envelope gene sequences used in the genotype phylogenetic analysis.

Genotype	GenBank accession number	Strain	Local of isolation	Year of isolation
Genotype 1	KU509258	DENV12522	Eritrea	2010
Genotype 1	KX056474	DENV1/China/YN/15DGR4102015	China	2015
Genotype 1	MH594897	NIV1740000NashikIndia2017	India	2017
Genotype 1	MK506262	hawaii2007	Thailand	2007
Genotype 1	Q868637	DENV1/IPC/BIDV3919/2000	Cambodia	2000
Genotype 2	AF425629	254363	Thailand	1963
Genotype 2	D10513	TH-SMAN	Thailand	1954
Genotype 2	JF297570	606147	Thailand	1960
Genotype 2	JQ922547	DENV1/THAI/606147/1960	Thailand	1960
Genotype 2	KC589010	SMGSE003	Indonesia	2012
Genotype 3	EF457905	P721244	Malaysia	1972
Genotype 3	FN825674	D1/Malaysia/36046/05	Malaysia	2005
Genotype 4	KY275197	D1/Timor/804/2010	EastTimor	2010
Genotype 4	MG894909	D1/Indonesia/1602aTw	Taiwan	2016
Genotype 4	MG894962	D1/Indonesia/1611aTw	Taiwan	2016
Genotype 4	MH634401	US/DB202/2001	USA	2001
Genotype 4	MH921567	PNG 2016	Australia	2016
Genotype 5	KF184975	Angola2013	Angola	2013
Genotype 5	MG877557	Gabon2012	Gabon	2012
Genotype 5	MH401998	KCMM25/BR/MS/2016	Brazil	2016
Genotype 5	MH679991	SGEHID1/50613Y14	Singapore	2013
Genotype 5	MK039555	US/DB225/2013	Puerto Rico	2013
Genotype 5		273756	Medina Gounass - Senegal	2015
Genotype 5		297855	Mali	2017
Genotype 5		297914	Louga - Senegal	2017
Genotype 5		297918	Louga - Senegal	2017
Genotype 5		297919	Louga - Senegal	2017
Genotype 5		298272	Louga - Senegal	2017
Genotype 5		302883	Louga - Senegal	2017
Genotype 5		303165	Louga - Senegal	2017
Genotype 5		307339	Louga - Senegal	2017
Genotype 5		307419	Louga - Senegal	2017
Genotype 5		307424	Louga - Senegal	2017
Genotype 5		310386	SaintLouis - Senegal	2018
Genotype 5		310460	Dakar - Senegal	2018
Genotype 5		313165	Fatick - Senegal	2018
Genotype 5		313167	Fatick - Senegal	2018
Genotype 5		313169	Fatick - Senegal	2018
Genotype 5		313639	Fatick - Senegal	2018
Genotype 5		313644	Fatick - Senegal	2018
Genotype 5		313718	Fatick - Senegal	2018
Genotype 5		313852	Fatick - Senegal	2018
Genotype 5		313943	Fatick - Senegal	2018
Genotype 5		313990	Fatick - Senegal	2018
Genotype 5		313997	Fatick - Senegal	2018
Genotype 5		314074	Fatick - Senegal	2018

Genotype 5		314343	Fatick - Senegal	2018
Genotype 5		314438	Fatick - Senegal	2018
Genotype 5		316359	Diourbel - Senegal	2018
Genotype 5		316382	Diourbel - Senegal	2018
Genotype 5		316413	Diourbel - Senegal	2018
Genotype 5		316447	Diourbel - Senegal	2018
Genotype 5		316474	Diourbel - Senegal	2018
Genotype 5		316548	Diourbel - Senegal	2018
Genotype 5		316647	Diourbel - Senegal	2018
Genotype 5		316649	Diourbel - Senegal	2018
Genotype 5		318267	Louga - Senegal	2019
Genotype 5		318479	Louga - Senegal	2019

Table S2. Dengue virus 1 Envelope gene sequences used in the phylogeographic and phylodynamics analysis.

Genotype	GenBank accession number	Strain	Local of isolation	Year of isolation
Genotype 5	KF184975	Angola2013	Angola	2013
Genotype 5	KM277613	AO/DB135/2013	Angola	2013
Genotype 5	KM277610	AO/DB132/2013	Angola	2013
Genotype 5	KM277612	AO/DB134/2013	Angola	2013
Genotype 5	KT825063	Cairns2014c	Australia	2014
Genotype 5	KT825067	Cairns2015	Australia	2015
Genotype 5	JN036371	BDH809/DENV1	Bangladesh	2009
Genotype 5	KP849868	D1/Bhutan/P13085	Bhutan	2013
Genotype 5	KP849880	D1/Bhutan/P13139	Bhutan	2013
Genotype 5	KP849885	D1/Bhutan/P13152	Bhutan	2013
Genotype 5	KP849888	D1/Bhutan/P13156	Bhutan	2013
Genotype 5	KP849864	D1/Bhutan/P13064	Bhutan	2013
Genotype 5	EU179860	DS06210505	Brunei	2005
Genotype 5	KJ415094	ZJ/03/13	China	2013
Genotype 5	MG560224	P1058/China/GD/GZ/2014	China	2014
Genotype 5	MG560223	P1057/China/GD/GZ/2014	China	2014
Genotype 5	KF864667	Zj/yw01	China	2013
Genotype 5	KT453243	FSD14020	China	2014
Genotype 5	DQ285562	Comoros04.329/93	Comoros	1993
Genotype 5	MG877557	Gabon2012	Gabon	2012
Genotype 5	KF289073	P23086	India	1956
Genotype 5	MG721063	R1J10	India	2016
Genotype 5	MH594879	NIV1733212NashikIndia2017	India	2017
Genotype 5	MH594894	NIV1738925NashikIndia2017	India	2017
Genotype 5	JN415507	India2008	India	2008
Genotype 5	KT825056	Indonesia2013b	Indonesia	2013
Genotype 5	LC038147	D1/Hu/Indonesia/NIID75/2014	Indonesia	2014
Genotype 5	KJ806818	MYSSelangorD1/5124Y12	Malaysia	2012
Genotype 5	KT175087	D1/Malaysia/1408aTw	Malaysia	2014
Genotype 5	KT175089	D1/Malaysia/1412bTw	Malaysia	2014
Genotype 5	KU666943	TM242	Malaysia	2014
Genotype 5	LC038145	D1/Hu/Malaysia/NIID64/2014	Malaysia	2014
Genotype 5	KY495795	Maldives2016	Maldives	2016

Genotype 5	KT825026	Maldives2014	Maldives	2014
Genotype 5	JF800928	DbH2010	Nepal	2010
Genotype 5	JF754981	NBH2910	Nepal	2010
Genotype 5	JF754982	NBPH1210	Nepal	2010
Genotype 5	JF754980	NBH1210	Nepal	2010
Genotype 5	AM746218	945	Saudi Arabia	1994
Genotype 5	AM746219	841	Saudi Arabia	1994
Genotype 5		297855	Mali	2017
Genotype 5		314438	Fatick - Senegal	2018
Genotype 5		316474	Diourbel - Senegal	2018
Genotype 5		318479	Louga - Senegal	2019
Genotype 5		316447	Diourbel - Senegal	2018
Genotype 5		316548	Diourbel - Senegal	2018
Genotype 5		316649	Diourbel - Senegal	2018
Genotype 5		310386	SaintLouis - Senegal	2018
Genotype 5		316413	Diourbel - Senegal	2018
Genotype 5		316647	Diourbel - Senegal	2018
Genotype 5		310460	Dakar - Senegal	2018
Genotype 5		316382	Diourbel - Senegal	2018
Genotype 5		316359	Diourbel - Senegal	2018
Genotype 5		318267	Louga - Senegal	2019
Genotype 5		307424	Louga - Senegal	2017
Genotype 5		307419	Louga - Senegal	2017
Genotype 5		297918	Louga - Senegal	2017
Genotype 5		298272	Louga - Senegal	2017
Genotype 5		297914	Louga - Senegal	2017
Genotype 5		297919	Louga - Senegal	2017
Genotype 5		302883	Louga - Senegal	2017
Genotype 5		303165	Louga - Senegal	2017
Genotype 5		273756	Medina Gounass - Senegal	2015
Genotype 5		307339	Louga - Senegal	2017
Genotype 5		313639	Fatick - Senegal	2018
Genotype 5		313165	Fatick - Senegal	2018
Genotype 5		313167	Fatick - Senegal	2018
Genotype 5		313169	Fatick - Senegal	2018
Genotype 5		313644	Fatick - Senegal	2018
Genotype 5		313718	Fatick - Senegal	2018
Genotype 5		313852	Fatick - Senegal	2018
Genotype 5		313943	Fatick - Senegal	2018
Genotype 5		313997	Fatick - Senegal	2018
Genotype 5		314074	Fatick - Senegal	2018
Genotype 5		314343	Fatick - Senegal	2018
Genotype 5		313990	Fatick - Senegal	2018
Genotype 5	GQ357690	SGEHIDED06807	Singapore	2007
Genotype 5	JN544409	SGEHID1/09106Y11	Singapore	2011
Genotype 5	GQ357692	SGEHIDED65008	Singapore	2008
Genotype 5	MF033212	50458	Singapore	2013
Genotype 5	MH679991	SGEHID1/50613Y14	Singapore	2013
Genotype 5	EF654104	DenKor01	SouthKorea	2004
Genotype 5	FJ687475	DenKor08	SouthKorea	2006

Genotype 5	JN415524	SriLanka2004	SriLanka	2004
Genotype 5	MG894702	D1/Malaysia/1205aTw	Taiwan	2012
Genotype 5	MG894694	D1/India/1110bTw	Taiwan	2011
Genotype 5	MG894919	D1/Singapore/1606aTw	Taiwan	2016
Genotype 5	MG894946	D1/Malaysia/1609aTw	Taiwan	2016
Genotype 5	MG894764	D1/Singapore/1306aTw	Taiwan	2013
Genotype 5	AY732379	ThD1003883	Thailand	1983
Genotype 5	AY732447	ThD1067380	Thailand	1980
Genotype 5	AY732429	ThD1044280	Thailand	1980
Genotype 5	AY732411	ThD1012780	Thailand	1980
Genotype 5	KT825042	Thailand2015a	Thailand	2015

Table S3. Model comparison of strict molecular clock and demographic growth models through path sampling (PS) and stepping stone (SS) methods. Bold numbers indicate the best fitting model.

	**Relaxed molecular clock**	
**Demographic growth model**	**PS**	**SS**
**Bayesian skyline plot**	**-5921.05**	**-5921.23**
Bayesian skyride plot	-5964.50	-5965.67
Bayesian skygrid model	-5932.60	-5932.83

PS: Path sampling; SS: Stepping stone.
